# Integrated Wearable System for Monitoring Skeletal Muscle Force of Lower Extremities

**DOI:** 10.3390/s24144753

**Published:** 2024-07-22

**Authors:** Heng Luo, Ying Xiong, Mingyue Zhu, Xijun Wei, Xiaoming Tao

**Affiliations:** 1 Research Institute for Intelligent Wearable Systems, The Hong Kong Polytechnic University, Hong Kong SAR 999077, China; henryheng.luo@connect.polyu.hk (H.L.); ying-xy.xiong@connect.polyu.hk (Y.X.); mingyue-aurora.zhu@connect.polyu.hk (M.Z.); 2 School of Fashion and Textiles, The Hong Kong Polytechnic University, Hong Kong SAR 999077, China; 3 Department of Rehabilitation Medicine, Shenzhen Hospital, Southern Medical University, Shenzhen 518100, China

**Keywords:** capacitive pressure sensor, integrated smart compression stocking system, lower extremities, maximum voluntary isometric contraction, muscle force, systemic design, two-way ANOVA

## Abstract

Continuous monitoring of lower extremity muscles is necessary, as the muscles support many human daily activities, such as maintaining balance, standing, walking, running, and jumping. However, conventional electromyography and physiological cross-sectional area methods inherently encounter obstacles when acquiring precise and real-time data pertaining to human bodies, with a notable lack of consideration for user comfort. Benefitting from the fast development of various fabric-based sensors, this paper addresses these current issues by designing an integrated smart compression stocking system, which includes compression garments, fabric-embedded capacitive pressure sensors, an edge control unit, a user mobile application, and cloud backend. The pipeline architecture design and component selection are discussed in detail to illustrate a comprehensive user-centered STIMES design. Twelve healthy young individuals were recruited for clinical experiments to perform maximum voluntary isometric ankle plantarflexion contractions. All data were simultaneously collected through the integrated smart compression stocking system and a muscle force measurement system (Humac NORM, software version HUMAC2015). The obtained correlation coefficients above 0.92 indicated high linear relationships between the muscle torque and the proposed system readout. Two-way ANOVA analysis further stressed that different ankle angles (*p* = 0.055) had more important effects on the results than different subjects (*p* = 0.290). Hence, the integrated smart compression stocking system can be used to monitor the muscle force of the lower extremities in isometric mode.

## 1. Introduction

Skeletal muscles, as the prime movers in the human musculoskeletal system, have garnered substantial interest from athletes, coaches, doctors, therapists, and researchers alike [1,2]. The lower extremity muscles are vital for many activities, including walking, running, jumping, and climbing stairs [3,4], and are also essential for maintaining balance and posture [5]. The gastrocnemius muscle is a large and powerful muscle located in the back of the lower leg. It is responsible for plantar flexing the foot (pointing the toes down) and flexing the knee. The gastrocnemius muscle is also a powerful shock absorber, helping to protect the joints from injury when the feet strike the ground. In conjunction with the deeper soleus muscle, this group is referred to as the triceps surae, which contribute approximately 80% of the force of plantarflexion [6,7].

Despite the gastrocnemius muscle’s substantial role in fundamental postural and locomotive functions, research directly examining the biomechanical mechanisms of in vivo muscle force generation during its contraction is scarce. A primary challenge is the absence of precise, concurrent quantitative instruments for dynamically assessing muscle status in relation to the resultant muscle force. Conventional modeling approaches, including electromyography (EMG)-driven and physiological cross-sectional area (PCSA)-driven models, encounter intrinsic difficulties in acquiring accurate and simultaneous data.

EMG is a well-established non-invasive technique for recording muscle electrical activity and estimating muscle force [8]. Guidelines such as SENIAM have been developed to standardize this method [9,10]. Nonetheless, the accuracy of EMG is compromised by electrical cross-talk [11], particularly as muscle strength recordings increase. This interference is pronounced in small muscle regions when surface electrodes are placed near adjacent muscles. Cross-talk is further complicated by factors such as tissue homogeneity, muscle tissue anisotropy, and the complexity of deep tissue structures, making it difficult to isolate signal contamination sources.

The variations in electrode locations cause reproducibility issues. These variations are influenced by muscle fiber distribution, the relative positions of fast and slow fibers, and their proximity to the electrodes. The amplitude of action potentials correlates with the anatomical diameter of muscle fibers, with fast fibers producing larger amplitudes than slow fibers. The signal amplitude may be diminished if electrodes are positioned farther from active fibers, underscoring the importance of the location and distribution of fast fibers relative to the electrodes in the integrity of surface EMG signals [12]. 

The quality of EMG data can be further reduced by various factors on an individual level, including random electrical noise, electromagnetic disturbance, electrode misplacement, intra-subject variation, and experimental discrepancy [13]. In summary, the EMG method is prone to influence by cross-talk, variations in electrode locations, involvement of synergistic muscles related to force generation, etc. [14].

On the other hand, the PCSA serves as an alternative representative measure of a muscle’s force capacity [15], which is a function of muscle volume, fascicle length, and pennation angle. To estimate the muscle performance through the maximum isometric force or instantaneous muscle power, the corresponding result is obtained by the PCSA multiplied by the maximum isometric stress [16,17,18]. In practice, the PCSA can be measured to determine the cross-sectional area of a muscle at a specific point along its length by using a variety of approaches [19]. Techniques such as magnetic resonance imaging (MRI) and computed tomography (CT) offer non-invasive means to capture detailed soft tissue images, while ultrasound utilizes sound waves for similar purposes. Despite their utility, these techniques are often associated with limitations, including significant expense, time-consuming processes, limited accessibility in smaller medical facilities, cumbersome equipment, reduced portability, inconvenient usage, and, in some cases, potential radiation exposure risks to patients. Consequently, these techniques fall short of providing a wearable, user-friendly, and real-time measurement solution.

To address the identified limitations, Smart Textile Integrated Microelectronic Systems (STIMES) [20] present a viable approach. The rapid advancement of STIMES has heightened interest in fabric-based sensors, notably those using capacitance [21,22,23,24], piezoresistivity [25,26], piezoelectricity [27,28,29], and triboelectricity [30,31,32,33]. Piezoelectric and triboelectric sensors are of particular interest due to their self-powering capability. However, these sensors depend on dynamic movement stimuli for signal generation, causing the output voltage to drift under sustained pressure, making them suitable primarily for dynamic pressure measurements. Conversely, piezoresistive and capacitive sensors are adept at both static and dynamic pressure measurements. Piezoresistive sensors are promising for wearable applications due to their simplicity, cost-effectiveness, flexibility, and versatility. Capacitive sensors, as alternatives, offer high sensitivity, low power consumption, stability, durability, low hysteresis, and rapid response. Moreover, Ref. [34] emphasizes the importance of encapsulation materials in mitigating mechanical, thermal, and electrical modes of sensor failure.

Previous studies [1,2] have explored upper arm muscle force by applying fabric-resistive strain sensors to detect the limb circumferential strain and further predict the muscle force using the circumference-to-torque model in isometric, isokinetic, and isotonic modes. This approach has demonstrated continuous monitoring, comfort, portability, and suitability for sports applications. However, it has not been extended to lower extremity muscles, which are critically involved in weight-bearing and locomotion. Additionally, each fabric-based sensor type possesses distinct advantages and characteristics. Resistive sensors suffer from nonlinearity due to inhomogeneous structural changes under strain, whereas capacitive sensors exhibit superior linearity. Consequently, the existing circumference-to-torque model is not universally applicable and requires further investigation.

On the other hand, Natural User Interface (NUI) [35,36] has become more popular, which stresses building a user-friendly bridge between the physical world and the digital world. The current literature reveals a lack of comprehensive research on the systemic design of end-to-end user-centered STIMES NUI research, particularly within the healthcare sector where there is a critical need for accessibility, cost-effectiveness, sustained engagement, efficient patient-doctor communication, and effective follow-up care. Addressing these gaps, this paper proposes an integrated smart compression stocking system designed to provide healthcare services to anyone, anytime, anywhere.

This system is particularly innovative in its ability to detect physiological changes during muscle activity. Specifically, it monitors the gastrocnemius muscle, in which an increase in muscle volume and associated morphological changes occur concurrently with muscle contraction. The proposed wearable system is capable of detecting these changes. The objective of this study is to explore the correlation between muscle force and muscle morphological changes, as detected by this novel system. This research aims to contribute to the development of more effective, low-cost, promptly responsive, non-invasive wearable healthcare technologies.

## 2. Materials and Methods

### 2.1. Pipeline Architecture Design

**Figure 1:**
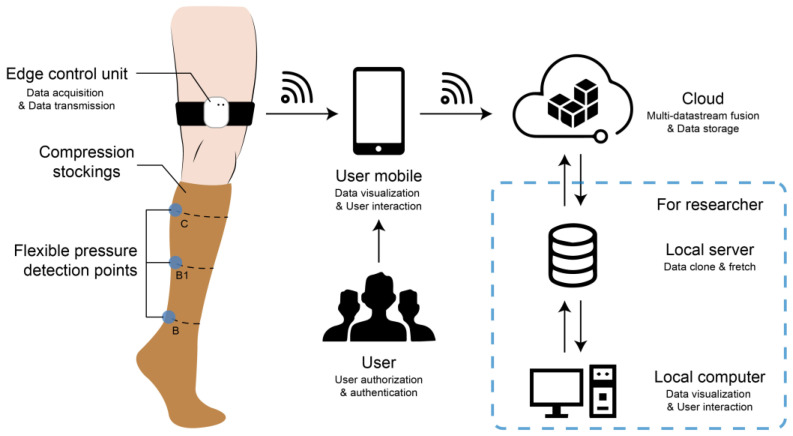
Pipeline architecture design for the integrated smart compression stocking system, including compression garments, fabric-embedded pressure sensors, an edge control unit, a user mobile application, and cloud backend.

This paper contemplated an integrated smart compression stocking system, which consisted of compression garments, fabric-embedded pressure sensors, an edge control unit, a user mobile application, and cloud backend. This system takes advantages of static and dynamic pressure responses, high pressure sensitivity, satisfactory accuracy, flexibility, user-friendliness, easy external integration, fast internal upgrades and repairs, and easy adaptation to various application scenarios. The pipeline architecture design is presented in Figure 1.

Laboratory-fabricated textile capacitive pressure sensors with a sandwich structure possess the benefits of facile fabrication, satisfactory accuracy, high repeatability, high sensitivity within the low-pressure range below 50 mmHg, low power consumption, and a fast response. The flexible sensors were located at the B point, B1 point, and C point of interest of compression stockings according to the RAL-GZ 387/1 standard [37]. The functions of electrical signal detection, processing, and wireless transmission were implemented by the proposed edge control unit with a low cost and low power consumption. An Android mobile phone application was developed to visualize data, enhance the natural user interface, and communicate streaming data with the cloud backend. The cloud backend implemented multi-user data storage, authentication and authorization, a datacenter dashboard, continuous integration, and continuous delivery.

### 2.2. Fabric Sensor Material and Fabrication

The design of the capacitive pressure sensor involved the implementation of a sandwich structure, wherein two layers of conductive fabric served as electrodes, with a dielectric layer positioned between them. The dielectric layer was constructed using a combination of polydimethylsiloxane (PDMS) and carbon black (CB) powder, which underwent additional processing with abrasive paper.

The sensors were encapsulated by protective layers of thermoplastic polyurethane (TPU) to reinforce their resistance and durability against external friction and strain. The flexible capacitive pressure sensors can be attached to various positions on compression stockings. In this study, the sensor was attached to the B point, B1 point, and C point on the stockings. The performance of the flexible sensors was tested, including sensitivity, hysteresis, repeatability, and washability tests. The sensors’ performance was assessed and characterized using an Instron 5944 testing machine (Instron, Norwood, MA, USA) equipped with a loading apparatus. Additionally, an LCR meter (E4980A, Keysight Technologies, Santa Rosa, CA, USA) was employed to measure and analyze the capacitance properties of the sensors. The load pressure and the capacitance measurements were obtained simultaneously.

**Figure 2:**
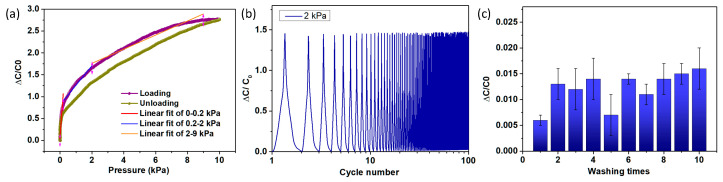
Performance of fabric capacitive pressure sensors. (**a**) Sensitivity and hysteresis testing. (**b**) Repeatability testing. (**c**) Washability testing.

The initial capacitance without any external loading was measured to be around 16 pF. As shown in Figure 2a, the sensitivity of the sensors is 3.44 kPa^−1^ for a loading pressure range of 0–0.2 kPa, 0.38 kPa^−1^ for a range of 0.2–2 kPa, and 0.19 kPa^−1^ for a range of 2–9 kPa. The upscale and downscale loading approaches to the same pressure point were utilized, and the largest difference was calculated accordingly, as denoted in Figure 2a, in which the hysteresis is approximately 8% at 4 kPa pressure. Cyclic pressure and release were conducted under a condition of 2 kPa pressure with a frequency of 0.1 Hz. Figure 2b shows the sensors’ repeatability characteristics, in which the capacitance values are stable during 100 cycles. According to the AATCC LP2 standard [38], the sensor’s capacitance was measured before and after hand washing, and was repeated 10 times. The results indicated that the sensor remained intact following 10 cycles of hand washing, exhibiting no significant capacitance alterations. Specifically, the capacitance variation remained within a 2% margin through the 10 washing cycles, as depicted in Figure 2c. 

### 2.3. Edge Control Unit Design

Instead of the prescribed therapeutic treatment of patients, the class I compression garment (RAL-GZ 387/1 standard) was used for healthy subjects in this study, in which less than 18 mmHg of pressure was used for less of an environmental effect on muscles and higher long-term wear conformity. An edge control unit should dynamically measure the sensors’ capacitance values and wirelessly transmit real-time data. The principal design factors comprised safety, low power consumption, low cost, long-term sustainable use, and portability. Thus, all components were selected from off-the-shelf commercial, popular electronic components, in which the main controller chip utilized in the system was a STM32 (STMicroelectronics, Geneva, Switzerland), the capacitance Analog-to-Digital Converter was a PCap01 (Sciosense B.V., Eindhoven, The Netherlands), the Bluetooth functionality was facilitated by an nRF51802 processor (Nordic Semiconductor, Trondheim, Norway), the charging control was managed by a TP4059 chip (Top Power ASIC Corp., Nanjing, China), and a 2400 mAh lithium battery pack was used. The bill of material (BOM) cost of the edge control unit was estimated as 234 RMB or 33 USD, as demonstrated in Table 1.

The capacitance values of the textile sensors, ranging from 0 to 100 pF, required the use of PCap01Ax-0301 chips for their high precision in detecting minute capacitance fluctuations. Each PCap01 chip facilitated three-channel capacitance measurements. The STM32F103C8T6 microcontroller was employed to manage the power supply and facilitate data transmission. Capacitance data, captured as a time series from the PCap01, were conveyed to the STM32 via a Serial Peripheral Interface (SPI) and then relayed to the nRF51802 through the SPI. The nRF51802 module was tasked with the wireless transmission of data to user mobile devices using Bluetooth Low Energy (BLE) technology.

**Figure 3:**
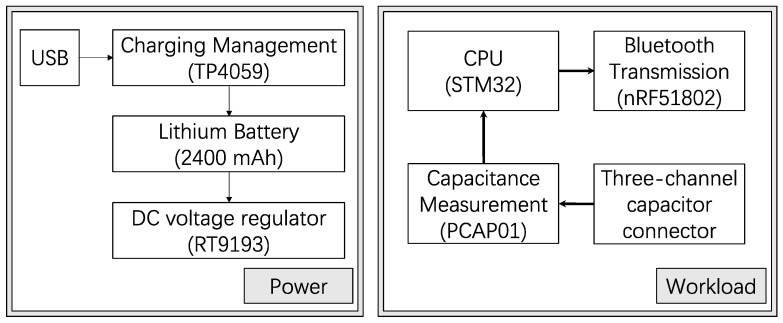
Schematic diagram of the edge control unit design.

For power management, the TP4059 chip was selected for its ability to charge a lithium battery via a Universal Serial Bus (USB). The charging status was indicated by two LEDs: green for charged and red for charging. A low-dropout regulator RT9193 (Richtek Technology Corp, Hsinchu, Taiwan) converted the lithium battery’s output to a stable 3.3 V to satisfy the circuit’s power requirements. A 2400 mAh lithium battery pack was chosen to guarantee daily functionality, enabling up to 8 h of continuous operation for three consecutive days. The functional schematic diagram is presented in Figure 3. 

A 63 × 45 mm 2-layer printed circuit board (PCB) was selected for its compactness, cost-effectiveness, efficient design, and resistance to interference. The housing was fabricated using advanced 3D printing techniques, featuring chamfered edges to reduce the risk of injury to users. The final housing, with dimensions of 68.50 × 21.70 × 51.05 mm, was made from acrylonitrile butadiene styrene (ABS), a material commonly used for its durability.

### 2.4. User Interface and Cloud Backend Design

Commercial smartphones were chosen as the primary interface for user interaction with the system, offering a suite of functionalities, such as information display and processing, danger alerts, message notifications, and practice recommendations. The smartphone stores data from the edge control unit in an SQLite database, with the smartphone handling heavy computational tasks and data storage to reduce the edge control unit’s resource and power consumption.

Considering the prevalent use of Android smartphones, the “Smart Compression Stocking Utility version 1” application was developed in Android Studio 4.1 using Java, targeting devices using Android 8.0 or higher. The application’s user interface facilitates login, Bluetooth device scanning and pairing, and data visualization. Users begin by registering or logging in, followed by scanning for and connecting to Bluetooth-enabled smart compression stockings. The application utilizes Android 8.0’s architecture and the Bluetooth protocol, along with socket methods and Android’s native binder, to ensure a stable connection and efficient data communication between mobile devices and edge control units. This enables the display of three-channel capacitance data in formats such as graphs, text, and analytical reports.

**Figure 4:**
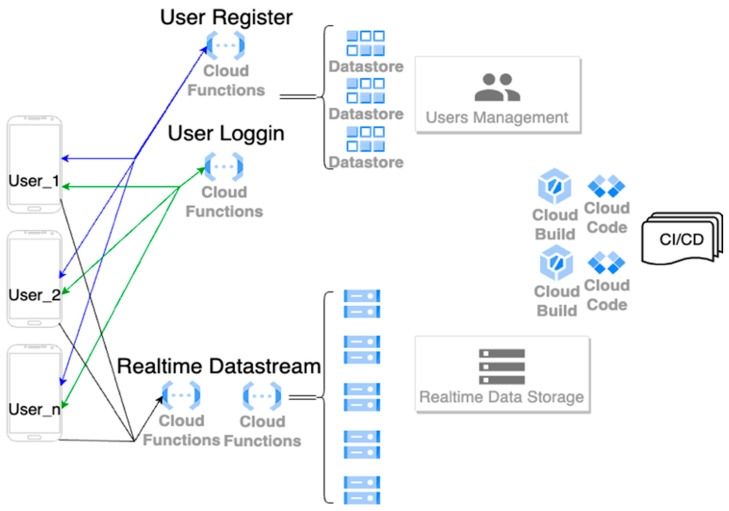
Cloud backend architecture. Services for multi-user account authorization (register and login) and user pressure time-series data streaming were developed and deployed on the cloud serverless microservices.

Furthermore, to provide convenient use for anyone, anywhere, anytime, a cloud backend was designed and deployed. The cloud backend takes advantages of instantaneously scaling up and down the computing capacity and storage according to demand, automatically and flexibly adjusting the resource allocation, fast time response, secure data communication, and cost savings, and is easy to test and deploy globally. Google Cloud Platform (GCP) was chosen to demonstrate the feasibility of the data processing pipeline. The Android application Smart Compression Stocking Utility was upgraded to version 2 and communicated with the deployed cloud endpoint through the Hypertext Transfer Protocol Secure protocol (HTTPS). Using the microservice architecture to achieve functional decoupling and flexible service scaling, the data streaming and user account management were developed by using Node.js and JavaScript programming languages to code and deploy on GCP serverless functions. In this way, user information data (such as account name, password, email, phone, age, gender, weight, height) and user real-time measurement data were stored in the cloud SQL databases. Relevant code was stored, built, and integrated in the cloud to achieve continuous integration and continuous delivery (CI/CD). The cloud backend architecture is illustrated in Figure 4. 

Particularly for the convenience of following clinical experiment data collection, the Android application Smart Compression Stocking Utility was upgraded to version 3 to add a new event trigger function, so that the “Start Exercise” button on the application user interface could be clicked to start recording the capacitance value data stream.

## 3. Experiment and Data Collection

### 3.1. Subjects and Experimental Protocol

The clinical study involved a cohort of twelve physically fit adult subjects, including six males and six females, ranging in age from 21 to 33 years, in height from 150 cm to 190 cm, and in weight from 43 kg to 77 kg, as shown in Table 2, in which exclusion criteria encompassed several factors, namely a self-reported incapacity to perform safe transfers or sustain a seated position for over 30 min; a medical history involving myopathy, sustained movement-induced ankle clonus or leg muscle spasms; past surgical interventions on the tested lower extremity targeting the leg or ankle; a documented history of moderate to severe brain injury; a history of spinal cord injury; known cases of cauda equina syndrome; the presence of radiculopathy; and a body mass index (BMI) exceeding 35. Before all subjects read and signed the informed consent document approved by the Hong Kong Polytechnic University Institutional Review Board, they were also informed and agreed to data privacy issues and experiment risks.

**Figure 5:**
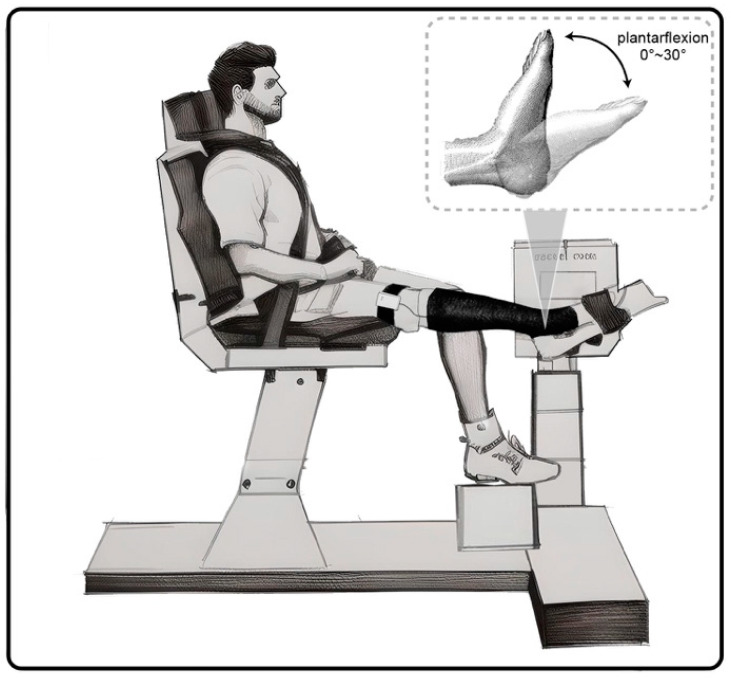
Experimental setup and exercise protocol. The subject’s right foot was to perform MVIC plantarflexion against the footplate and complete two sets of four exercises when the footplate was fixed at 0°, 10°, 20°, and 30°.

The subject was requested to wear a smart compression stocking on the right leg and adjust the location, ensuring one flexible sensor was on the C point (on the gastrocnemius belly where the largest cross-sectional area was observed). Subsequently, the subject was instructed to assume a stable seated position on a Humac NORM isokinetic dynamometer (CSMi, Stoughton, MA, USA), which served the purpose of accurately measuring and recording both the real-time plantarflexion position and the corresponding torque generated during plantarflexion, as demonstrated in Figure 5. The subject was positioned in an upright seated posture on a foam cushion, ensuring a hip flexion angle ranging from 100° to 120° to attain a comfortable and stable seating arrangement. The knee was maintained in a fully extended position (0° knee flexion), while the right leg ankle was kept in a neutral alignment. A waist belt was utilized to secure the subject firmly in place. The subject’ right foot was positioned in a flat orientation on the footplate and securely fastened dorsally using a padded Velcro strap. Furthermore, the foot was appropriately aligned with a fulcrum to ensure that the axis of the ankle overlapped the axis of the lever arm. This arrangement facilitated unrestricted ankle plantarflexion and dorsiflexion, enabling a full range of motion without encountering any impediments. 

The Humac NORM was prepared, calibrated, and configured to isometric mode in advance. Torque (NT)–time data was recorded for each experiment and each subject. A standby experiment operator clicked the “Start Exercise” button on Smart Compression Stocking Utility version 3 to start recording the capacitance value data stream, and simultaneously pressed the start button on the Humac NORM desktop software to start recording the torque data stream. Therefore, the capacitance value data stream and muscle torque data stream were synchronized. The edge control unit possessed a sampling rate of 10 Hz, while the Humac NORM possessed a sampling rate of 100 Hz. 

### 3.2. Test Protocol of Maximum Voluntary Isometric Contraction

During an isometric contraction of the muscle, the torque produced is considered a representative characteristic of skeletal muscle force. This holds true when the joint position remains constant, ensuring that the moment arm of the skeletal muscles also remains constant. Consequently, the generated torque serves as a quantifiable measure of the muscle force exerted. In addition, some researchers found that the maximum isometric force can be calculated by the PCSA multiplied by the maximum isometric stress [16,17,18]. Alternatively, other research focused on the linear relationship between the generated torque and limb circumference [1]. This research assumed linear relationships between the generated torque and pressure change from compression stockings caused by PCSA changes. 

**Figure 6:**
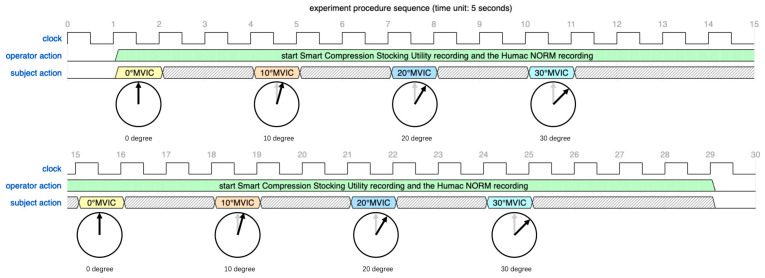
The clinical experiment procedure time sequence for each subject.

The testing protocol requested each subject to complete one set of four exercises and repeat. The Humac NORM lever arm was fixed at different positions of 0°, 10°, 20°, and 30° for different exercises, where 0° corresponds to the anatomic position and the direction of plantarflexion is considered positive. In each set, the subject was requested to perform a maximum voluntary isometric contraction (MVIC) against the fixed lever arm and hold it for 5 s. After resting for 10 s, the subject was encouraged to perform the next exercise for 5 s of MVIC. To avoid muscle fatigue and mitigate any discomfort, a rest duration of 20 s was prescribed between two sets. The test protocol time sequence is comprehensively illustrated in Figure 6.

### 3.3. Data Collection and Preprocessing

With the intent of finding the relationship between the pressure change (represented by the flexible capacitive sensors’ readout C) and muscle force (represented by the generated muscle torque values NT), we assumed there exists simple linearity, where fitting coefficients A and B are defined in Equation (1) and determined by the Least Squares (LS) method. Furthermore, Pearson’s correlation coefficients R between C and NT were calculated to evaluate the linear correlation, defined in Equation (2).

(1) NT=A×C+B

(2) $$R=\frac{conv(C,NT)}{\sigma (C)\times \sigma (NT)}$$

**Figure 7:**
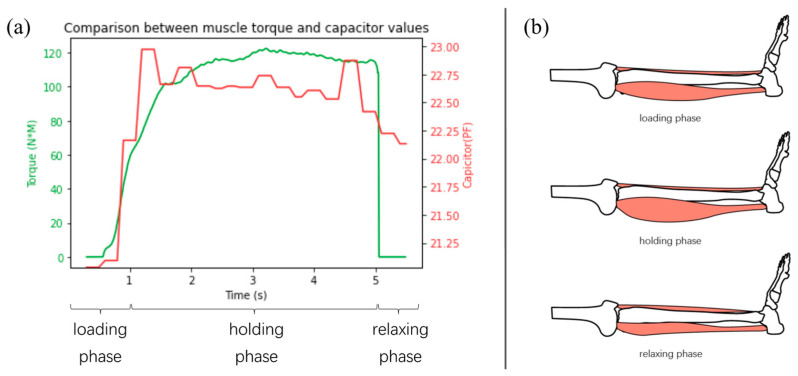
Illustrations of the MVIC’s different phases, consisting of the loading phase, the holding phase, and the relaxing phase. (**a**) A typical MVIC with measured torque values (NT) and capacitance values (C). (**b**) A typical gastrocnemius volume and morphologic change.

MVIC durations can be divided into distinct phases, namely the loading phase, the holding phase, and the relaxation phase, illustrated in Figure 7. The loading phase exhibits fine linearity between muscle volume changes and muscle force [1], whereas the holding phase and relaxation phase exhibit much more sophisticated skeletal muscles performance [39]. Hence, this research focused on the loading phase, which was predetermined as the first 1 s duration within each MVIC of eight different exercises for each subject.

To attenuate the impacts of differences in individual muscle performance and individual experimental discrepancies, regarding each subject, the raw time series data of muscle torque and capacitance were normalized by the same subject completing all assigned isometric exercises, which are defined in Equations (3) and (4). Furthermore, fitting coefficients α and β and Pearson’s correlation coefficients r were all calculated based on normalized capacitance values $C_{norm}$ and normalized torque values ${NT}_{norm}$, denoted by Equations (5) and (6).

(3) $${NT}_{norm}=\frac{NT-NT(start)}{\max\left(NT\right)-\min(NT)}$$

(4) $$C_{norm}=\frac{C-C(start)}{\max\left(C\right)-\min(C)}$$

(5) $${NT}_{norm}=\alpha \times C_{norm}+\beta$$

(6) $$r=\frac{conv(C_{norm},{NT}_{norm})}{\sigma (C_{norm})\times \sigma ({NT}_{norm})}$$

## 4. Results and Discussion

### 4.1. Data Analysis

**Figure 8:**
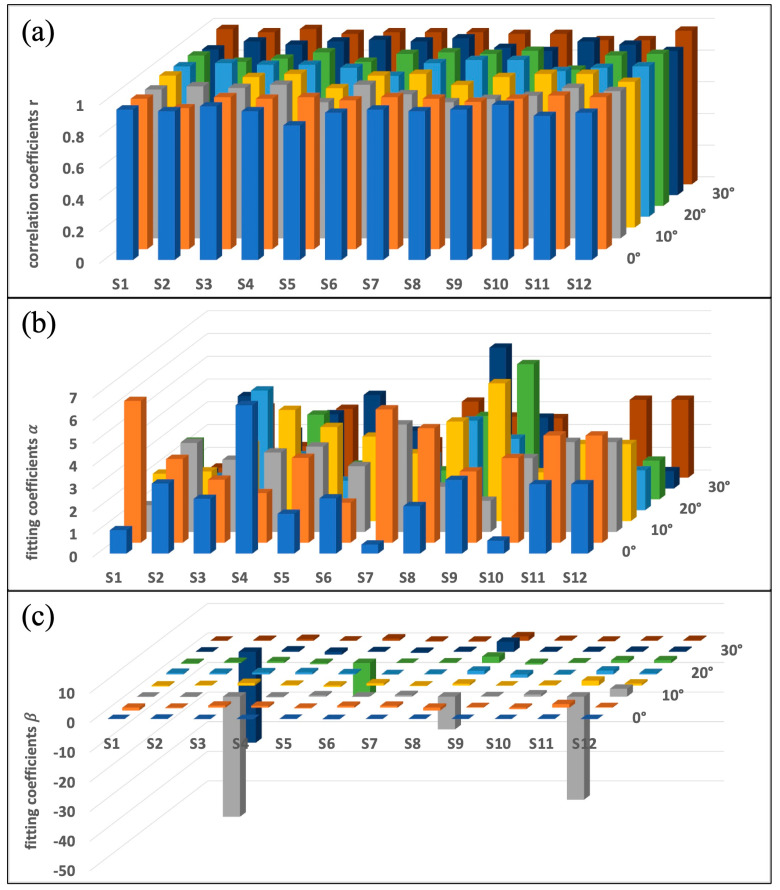
Illustration of fitting coefficients and correlation coefficients for each subject’s 8 MVIC exercises, where the *X*-axis represents 12 different subjects and the *Z*-axis represents two exercises at four different ankle angles of 0°, 10°, 20°, and 30°. (**a**) The correlation coefficient r for each MVIC exercise. (**b**) The fitting coefficient α for each MVIC exercise. (**c**) The fitting coefficient β for each MVIC exercise.

For each subject, all eight MVIC exercises were completed, incorporating two MVIC exercises at four different ankle positions of 0°, 10°, 20°, and 30°. The fitting coefficients α and β and correlation coefficients r between normalized capacitance values $C_{norm}$ and normalized torque values ${NT}_{norm}$ were calculated based on the loading phase of each MVIC exercise. The data visualization of all 12 subjects’ exercises is exhibited in Figure 8.

To further demonstrate, the mean and standard deviation of the fitting coefficients α and β and correlation coefficients r over all eight MVIC exercises for each subject were calculated and summarized in Table 3. For all subjects, the correlation coefficient mean was larger than 0.92, while the corresponding standard deviation was less than 0.08, which indicates the linear relationship between the normalized capacitances and normalized torques.

Moreover, a two-way Analysis of Variance (ANOVA) [1,40] was used to analyze the effects of two independent factors, different subjects and different ankle angles, on continuous dependent variables, which were the correlation coefficients r, the fitting coefficients α, and the fitting coefficients β, respectively. Two-way ANOVA was adopted, considering the ANOVA model is remarkably robust to violation of the normality assumption, which means that it will have a non-significant effect on Type I error rates, and *p* values remain reliable as long as there are no outliers. As the experiment involved twelve subjects and four ankle positions (0°, 10°, 20°, 30°), and in each situation two independent MVICs were performed and observed, the 12 × 4 balanced design was adopted.

We found no statistically significant difference in the effect on correlation coefficients r by either the subject factor (*p* = 0.233 > 0.05) or ankle position factor (*p* = 0.063 > 0.05), with no significant interaction effect between these two factors (*p* = 0.423 > 0.05). The result implies the general existence of linear relationships between the normalized capacitances and normalized torques, regardless of the effects of different subjects and different ankle positions.

We found no statistically significant difference in effect on coefficients α by either the subject factor (*p* = 0.290 > 0.05) or ankle position factor (*p* = 0.055 > 0.05), with no significant interaction effect between these two factors (*p* = 0.961 > 0.05). There was also no statistically significant difference in effect on coefficient β by either the subject factor (*p* = 0.821 > 0.05) or ankle position factor (*p* = 0.299 > 0.05), with no significant interaction effect between these two factors (*p* = 0.575 > 0.05).

**Figure 9:**
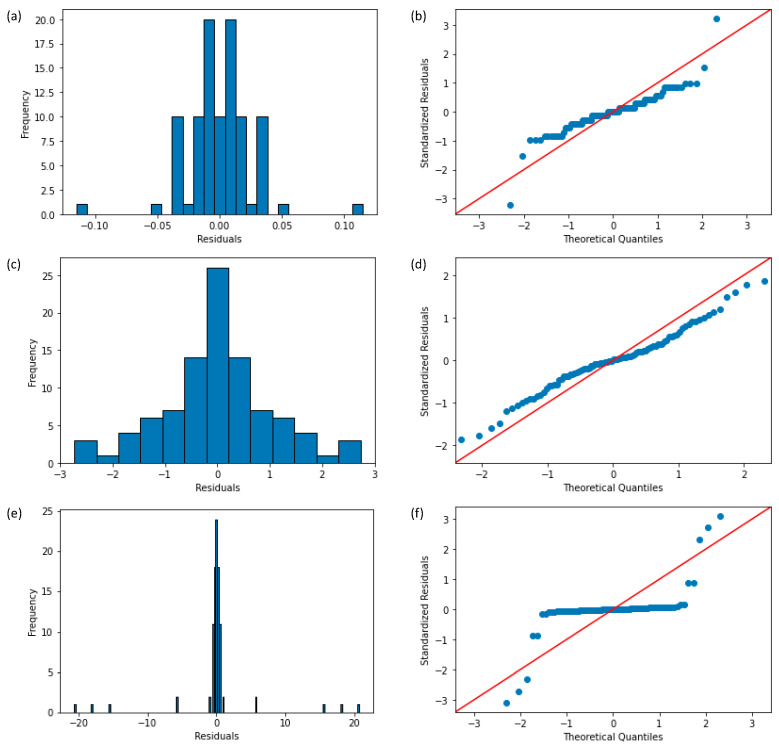
Visual approaches to test the ANOVA assumption of normality. Regarding the correlation coefficients r: the (**a**) histogram of residuals and (**b**) QQ-plot from standardized residuals; regarding the fitting coefficients α: the (**c**) histogram of residuals and (**d**) QQ-plot from standardized residuals; regarding the fitting coefficients β: the (**e**) histogram of residuals and (**f**) QQ-plot from standardized residuals.

Furthermore, considering the low sample size of 12 subjects, the assumption of normality, which represents residuals (experimental error) that are approximately normally distributed, was tested by histograms, QQ-plots (Quantile–Quantile Plots), and the Shapiro–Wilk test [41,42] for the correlation coefficients r, the fitting coefficients α, and the fitting coefficients β, respectively, for which graphs are displayed in Figure 9.

For the correlation coefficients r, although we reject the Shapiro–Wilk test statistics (*p* = 7 × 10^−7^ < 0.05), we further investigated the histogram and the residual plot. In the histogram, the distribution looks approximately normal and suggests that residuals are approximately normally distributed. As the standardized residuals lie around the 45-degree line, this suggests that the residuals are approximately normally distributed. For the fitting coefficients α, as the *p*-value of the Shapiro–Wilk test is non-significant (*p* = 0.169 > 0.05), we fail to reject the null hypothesis and conclude that data is drawn from a normal distribution. In the histogram, the distribution looks approximately normal and suggests that residuals are approximately normally distributed. As the standardized residuals lie around the 45-degree line, this suggests that the residuals are approximately normally distributed. For the fitting coefficients β, although we reject the Shapiro–Wilk test statistics (*p* = 8 × 10^−17^ < 0.05), we should further investigate the histogram and the residual plot. The histogram shows the approximately normal distribution of residuals. In the residual plot, standardized residuals lie approximately along the 45-degree line, which suggests that the residuals are approximately normally distributed.

We addressed the validity of the aforementioned results by the effective sample size calculations [1,43]. When the significance level alpha was chosen as 0.05 (the chance of a false-positive result, type I error), beta was chosen as 0.10 (the chance of a false-negative result, type II error) with a power of 0.90 (1-beta), assuming 0.90 or higher correlation coefficients accept the linear relationship (null hypothesis H_0_) while 0.80 or lower correlation coefficients reject the linear relationship (alternative hypothesis H_1_), and the correlation coefficients are approximately normally distributed with a standard deviation of 0.07, hence, the minimal sample size was derived equal to 11. After the clinical experiments, the mean standard deviation of the correlation coefficients r was calculated as 0.03, which represents that the assumed population standard deviation was larger than the real one (σ=0.07>0.03); therefore, the sample size was conservatively estimated. This a-priori sample size was satisfied by 12 subjects recruited in the research.

### 4.2. Discussion

The integrated smart compression stocking system was designed, implemented, and applied to monitor lower extremity muscle force. Nevertheless, the relationship between the normalized capacitances and normalized torques does not hold a perfect linear relationship. The plausible explanations are summarized. Firstly, there is complex deformation of the skeletal muscle during MVIC exercises. The triceps surae, including the two-headed (medial and lateral) gastrocnemius, the soleus, and the plantaris muscles can only generate 80% of the force of plantarflexion [6,7], and also deeper muscle deformation is hard to detect; therefore, it is satisfactory to consider the correlation coefficients achieved above 0.92. Secondly, although experimental settings were adjusted consistently for each subject, there still existed a discrepancy. When the subject performed MVIC exercises, since all requirements were informed verbally in advance, there were individual understanding and reaction discrepancies. Thirdly, to increase the flexible capacitive pressure sensors’ sensitivity, carbon black powder was mixed into PDMS, and the formed dielectric layer was roughened by abrasive paper. The sensor’s characteristics, such as the irregular microstructure in the dielectric layer and the viscoelastic property of PDMS, could cause the capacitance to change nonlinearly with respect to applied pressure.

The above factors also impact the fitting coefficients α and β. α is the slope of the linear relationship, serving as an indicator of the magnitude and proportionality of normalized torques determined by the normalized capacitances. From Table 3, its standard deviations for each subject are below 1.81, representing low dispersion over different ankle angle exercises for each subject. Although a two-way ANOVA indicates no statistically significant difference in effect on coefficients α by either subject factor (*p* = 0.290 > 0.05) or ankle position factor (*p* = 0.055 > 0.05), the ankle position factor plays a more important role, as its *p*-value is much smaller than that of the subject factor and closer to the predetermined popular threshold of 0.05. During clinical experiments, it was observed that the larger the ankle angle, the more difficulty a subject had generating muscle force. This can be verified by muscle force–length properties and other study data and observations [44,45,46]. Previous studies concluded that the intact human gastrocnemius muscle or soleus muscle operates in the ascending limb and plateau region of the bell-shaped force–length curve relationship.

β is the intercept of the linear relationship, serving as an indicator of the initial state or condition pertaining to each exercise within the context. Since it is impossible to passively adjust the accurate initial state of muscle contraction or relaxation for each subject, we performed a limited analysis of the fitting coefficients β; nevertheless, we noticed ubiquitous small values (approximate zero) with few exceptions. The obtained results from two-way ANOVA analysis of the correlation coefficients r and the fitting coefficients α were further verified by that of the fitting coefficients β.

The current research has the following limitations. Firstly, the flexible sensors exhibited limited sensitivity in subject clinical experiments. The capacitance changes achieved were 2 pF to 3 pF for most of the loading phases. While this sensitivity is sufficient to detect current muscle morphological changes, it is inadequate for capturing more nuanced variations. Hence, a higher sensitivity is desired. Secondly, the study recruited a total of twelve healthy young individuals as subjects, and clinical experiments were conducted in Hong Kong and Shenzhen due to time and cost constraints. Consequently, the applicability of the obtained results is confined to a limited scope, thereby impeding the universal application of the integrated smart compression stocking system. Thirdly, current research utilized the raw capacitance readout and accomplished pointwise linear regression. Nonetheless, the capacitance readout may be compromised by noise and fluctuations due to the laboratory-fabricated flexible capacitive pressure sensors. Despite achieving acceptable linearity results in the study, the system’s real-world applications in users’ daily lives require further consideration of complex conditions.

## 5. Conclusions and Future Work

The paper proposed an integrated smart compression stocking system, which includes compression garments, fabric-embedded pressure sensors, an edge control unit, a user mobile application, and cloud backend. The whole system was guided on an end-to-end user-centered STIMES design, presenting notable benefits of static and dynamic pressure detection, high sensitivity in the low-pressure range, adaptability, user-friendliness, low-cost, streamlined external integration, swift internal upgrade and repair capabilities, and seamless adaptability to diverse application scenarios.

Furthermore, the paper addressed the application of the system to detect the gastrocnemius muscle force during ankle plantarflexion in the maximum voluntary isometric contraction mode. The system was constructed and verified for linear relationships between the normalized capacitances of the system readout and normalized torques of the Humac NORM readout. Two-way ANOVA analysis implied that a linear relationship holds generally and that different ankle angles have more important effects on the results, rather than different subjects. 

In future research, our main aim is to generalize the findings and continuously improve the performance of the integrated smart compression stocking system. Firstly, we will explore a flexible sensor matrix, enhanced sensors, and a compression garment integration process to improve sensitivity. Secondly, we will recruit larger cohorts to increase the system’s persuasive power and generalizability. The inclusion of diverse or even contrasting subject samples is crucial for assessing the statistical significance of research findings. Thirdly, we will explore more dedicated data-driven machine learning algorithms to denoise and address outlier situations. Furthermore, the high-level feature extraction algorithms will contribute to greater clinical practice value, such as muscle atrophy and muscle hypertrophy detection, and falling down predictions.

## Figures and Tables

**Table 1:** The edge control unit BOM cost.

Module	Specification	Cost (RMB)
**Interface**	USB (Power Supply/wired data communication)	5
	Switch On/Off	1
	Reset	1
	SWD Debug port	1
	2 LED lights (Charging indicator)	4
	Capacitance Measure Interface (3 channels)	6
**Main Controller**	STM32	50
**Capacitance Measure**	PCap01	32
**Bluetooth** **Communication**	nRF51802	30
**Charging Controller**	TP4059	4
**LDO**	RT9193	2
**USB convert to serial interface**	ch340	3
**Battery**	Lithium Battery Pack (2400 mAh)	50
**PCB**	PCB (63 × 45 mm, 2 layers)	15
**Housing**	3D printing (68.50 × 21.70 × 51.05 mm)	30
**Total** **Cost**		234

**Table 2:** Physical characteristics of participant subjects.

Subject	Age (Years)	Gender	Height (cm)	Weight (kg)	BMI	Dominant Leg
Subject_1	25	M	173	65	21.7	R
Subject_2	21	M	175	65	21.2	R
Subject_3	21	M	170	60	20.8	R
Subject_4	21	F	162	45	17.1	R
Subject_5	22	F	166	77	27.9	R
Subject_6	21	F	165	75	27.5	R
Subject_7	22	F	151	44	19.3	R
Subject_8	21	F	150	43	19.1	R
Subject_9	22	F	155	47	19.6	R
Subject_10	31	M	190	75	20.8	R
Subject_11	33	M	175	65	21.2	R
Subject_12	26	M	174	66	21.8	R

**Table 3:** Summary of the coefficients (α and β) and correlation coefficients r for 12 subjects.

Subject	Correlation Coefficient Mean	Correlation Coefficient STD	Alpha Mean	Alpha STD	Beta Mean	Beta STD
Subject_1	0.95	0.02	1.99	1.73	−0.13	0.37
Subject_2	0.92	0.08	2.84	1.02	−3.70	10.20
Subject_3	0.96	0.01	2.85	1.08	−4.58	13.58
Subject_4	0.96	0.01	3.68	1.32	0.01	0.51
Subject_5	0.92	0.05	2.74	1.20	−1.27	3.79
Subject_6	0.95	0.03	2.27	0.72	0.07	0.38
Subject_7	0.96	0.02	2.76	1.75	0.18	0.32
Subject_8	0.94	0.04	3.74	1.37	−0.37	4.22
Subject_9	0.94	0.03	3.57	1.51	−0.09	0.46
Subject_10	0.93	0.04	2.11	0.97	0.09	0.34
Subject_11	0.94	0.02	2.84	1.24	−3.65	11.77
Subject_12	0.94	0.02	2.53	1.81	0.68	0.80

## Data Availability

The data used during the current study are available by the authors on request.
